# GRIM19 ameliorates acute graft-versus-host disease (GVHD) by modulating Th17 and Treg cell balance through down-regulation of STAT3 and NF-AT activation

**DOI:** 10.1186/s12967-016-0963-0

**Published:** 2016-07-08

**Authors:** Min-Jung Park, Seung Hoon Lee, Sung-Hee Lee, Eun-Kyung Kim, Eun Jung Lee, Young-Mee Moon, Mi- La Cho

**Affiliations:** The Rheumatism Research Center, Catholic Research Institute of Medical Science, The Catholic University of Korea, Seoul, South Korea; Divison of Rheumatology, Department of Internal Medicine, The Catholic University of Korea, Seoul, 137-040 South Korea; Conversant Research Consortium in Immunologic Disease, College of Medicine, The Catholic University of Korea, Korea 505 Banpo-Dong, Seocho-Ku, Seoul, 137-040 Korea

## Abstract

**Background:**

T helper (Th) 17 cells are a subset of T helper cells that express interleukin (IL)-17 and initiate the inflammatory response in autoimmune diseases. Regulatory T cells (Tregs) are a subpopulation of T cells that produce forkhead box P3 (FOXP3) and inhibit the immune response. Graft versus host disease (GVHD) is a complication of allogeneic tissue transplantation, and Th17 cells and their proinflammatory activity play a central role in the pathogenesis of GVHD. Gene associated with retinoid-interferon-induced mortality (GRIM) 19, originally identified as a nuclear protein, is expressed ubiquitously in various human tissues and regulate signal transducer and activator of transcription (STAT)3 activity.

**Methods:**

Splenoytes and bone marrow cells were transplanted into mice with GVHD. The alloresponse of T cells and GVHD clinical score was measured. Realtime-polymerase chain reaction (realtime-PCR) was used to examine mRNA level. Flow cytometry and enzyme linked immunosorbent assay (ELISA) was used to evaluate protein expression.

**Results:**

A GRIM19 transgenic cell transplant inhibited Th17 cell differentiation, alloreactive T cell responses, and STAT3 expression in mice with GVHD. On the other hand, the differentiation of Tregs and STAT5 production were enhanced by GRIM19. Overall, the severity of GVHD was decreased in mice that had received GRIM19 transgenic bone marrow and spleen transplants. Transplantation from GRIM19-overexpressing cells downregulated the expression of nuclear factor of activated T cells (NFATc1) but promoted the expression of regulator of calcineurin (RCAN)3 while downregulating NFAT-dependent cytokine gene expression. This complex mechanism underlies the therapeutic effect of GRIM19.

**Conclusions:**

We observed that GRIM19 can reduce Th17 cell differentiation and alloreactive T cell responses in vitro and in vivo. Additionally, GRIM19 suppressed the severity of GVHD by modulating STAT3 activity and controlling Th17 and Treg cell differentiation. These results suggest that GRIM19 attenuates acute GVHD through the inhibition of the excessive inflammatory response mediated by T cell activation.

## Background

Graft-versus-host disease (GVHD) is a condition induced by the release of excessive inflammatory cytokines. Donor-derived naive CD4^+^ T cells activated by alloantigens play an important role in the pathogenesis of GVHD. It has been demonstrated that GVHD is an immune inflammatory disease. It is a complication of bone marrow transplants. When donor-derived T cells differentiate into T helper (Th) cell subsets, they can produce unique sets of transcription factors and cytokines that can damage host tissues [1–3]. It has been suggested that Th1 cell alloresponses can induce transplant rejection. The progression of GVHD can be explained mainly by a Th1 response [4, 5]. However, Th17 cells and regulatory T cells (Tregs) have also been implicated in GVHD. It is well documented that Th17 cells can contribute to the severity of GVHD with the Th17/Treg ratio being higher in patients with GVHD [6]. Moreover, Th17 cell numbers are increased while Treg cell numbers are reduced in peripheral blood mononuclear cells of GVHD patients [7]. Recently, signal transducer and activator of transcription 3 (STAT3) has been described as an important regulator of Th17/Treg cells. Inhibition of STAT3 activation can reduce Th17 cell numbers but increase Treg numbers, thus attenuating inflammatory disorders [8, 9].

Gene associated with Retinoid-Interferon-induced Mortality (GRIM)19 is a 16-kDa protein primarily identified as a nuclear protein expressing constitutively in several human tissues. GRIM19 was first recognized as a factor involved in apoptosis [10]. GRIM19 also plays a role in inflammation because the expression of GRIM19 is diminished in inflamed mucosa of inflammatory bowel disease patients [11]. In addition, GRIM19 can interact with STAT3, a cytoplasmic transcription factor in Th17 cells. It has been suggested that GRIM19 can inhibit the transcriptional activity of STAT3 [12]. In addition, GRIM19 is involved in mitochondrial respiration and tumorigenesis via STAT3-responsive gene expression. Overexpression of GRIM19 has therapeutic properties against cancer by inhibiting STAT3-mediated signal transduction, while the absence of GRIM19 abrogates mitochondrial respiratory chain function and accelerates tumor development by enhancing the expression of STAT3-responsive genes [13–15]. Recently, GRIM19 has been suggested as a significant factor involved in Th17/Treg balance and STAT3 activation in inflammatory disease. Overexpression of GRIM19 can inhibit inflammation and improve collagen-induced arthritis by controlling the differentiation of Th17 cells and Tregs [16]. Here, we hypothesized that GRIM19 could reduce inflammatory response and regulate Th17/Treg balance in GVHD. To determine whether GRIM19 could attenuate GVHD, first we evaluated the role of GRIM19 in the alloreactive T cell response both in vivo and in vitro. We then determined the therapeutic function and anti-inflammatory activity of GRIM19 in vivo in a mouse model of GVHD. To understand how GRIM19 could decrease the inflammatory response, we analyzed its effect on Th17/Treg balance controlled by the STAT3 pathway in a GVHD mouse model.

## Methods

### Mice

C57BL/6 (B6, H-2 k^b^) and BALB/c (B/c, H-2k^d^) mice at 8–10 weeks of age were purchased from OrientBio (Sungnam, Korea). To generate GRIM19 transgenic mice, a pcDNA3.1+ (Invitrogen) vector was constructed containing CMV promoter. The GRIM19 fragment was synthesized by GenScript Corporation (NJ, USA), with codon optimization for expression in mammalian cells. The origin of open reading frame is mice. GRIM19 transgenic mice overexpresssing Grim19 were generated on a C57BL/6 background and maintained in facilities at (Macrogen Inc., Seoul, Korea) by microinjection of a transgene. GRIM19 transgenic mice founder transgenic mice were mated to C57BL/6 J mice. The presence of the transgene in the founders was confirmed by PCR using genomic DNA extracted from the tails of mice. GRIM19 transgenic mice were generated as previously described [16]. Mice were maintained under specific pathogen-free conditions in an animal facility with humidity of 55 ± 5 % and temperature of 22 ± 1 °C under 12/12-h of light/dark. The air in the facility was passed through a HEPA filter system to exclude bacteria and viruses. Animals were allowed ad libitum access to mouse chow and tap water. The protocols used in this study were approved by the Animal Care and Use Committee of the Catholic University of Korea.

### BMT model and histopathological scoring

Recipients B/c mice were intravenously injected with 5 × 10^6^ donor bone marrow cells after lethal irradiation with 700 cGy. Irradiated recipients received a single intravenous injection of WT or GRIM19 Tg splenotyces (1 × 10^7^ cells) through a lateral tail vein. Survival of mice after BMT was monitored daily. The extent of clinical GVHD was assessed weekly using a scoring system based on the following five clinical parameters: weight loss, posture, activity, fur texture, and skin integrity. Mice were euthanized on day 14 after BMT prior to blinded histopathology evaluation for GVHD target tissues such as skin, liver, small intestine, and large intestine [17]. Organs were harvested, cryo-embedded, and subsequently sectioned. Thsee sections were fixed in 10 % (v/v) buffered formalin and stained with hematoxylin and eosin (H&E) for histological examination.

### Alloreactive T cell responses in vitro

Splenocytes derived from B/c mice were used as “stimulator” cells in the context of allorecognition. Cells from B6 WT or GRIM19 Tg mice were used as “responder” cells. Splenocytes were harvested in ACK lysis buffer (0.15 M NH_4_Cl, 10 mM KHCO_3_, and 0.1 mM EDTA; pH 7.2–7.4), washed, and resuspended in complete culture medium (RPMI 1640 supplemented with 10 % [v/v] heat-inactivated fetal calf serum). Aliquots of 2 × 10^5^ CD4^+^ T cells (responders) were cultured with 2 × 10^5^ irradiated (2500 cGy) APCs in 96-well plates containing 200 µl/well of complete medium. Cells were incubated at 37 °C with a humidified 5 % (v/v) CO_2_/air atmosphere. Cells were pulsed with 1 µCi of tritiated thymidine (^3^[H]-TdR; NEN Life Science Products Inc., Boston, MA, USA) 18 h before harvesting and counted with an automated harvester (PHD Cell Harvester (Cambridge Technology, Inc., Cambridge, MA, USA). Results were expressed as mean cpm values of triplicate samples ± SD.

### Real-time polymerase chain reaction (PCR)

Total RNA was extracted using the TRI reagent (Molecular Research Center, Inc. Cincinnati, OH, USA) according to the manufacturer’s instructions. Complementary DNA was synthesized using SuperScript reverse transcription system (Takara). A Light-Cycler 2.0 instrument (software version 4.0; Roche Diagnostics) was used for PCR amplification. All reactions were performed using the LightCycler with FastStart DNA Master SYBR Green I mix (Takara) following the manufacturer’s instructions. The following primers were used to amplify mouse genes: *Il*-*17*, 5′-CCT-CAA-AGC-TCA-GCG-TGT-CC-3′ (sense) and 5′-GAG-CTC-ACT-TTT-GCG-CCA-AG-3′ (antisense); *Foxp3*, 5′-GGC-CCT-TCT-CCA-GGA-CAG-A-3′ (sense) and 5′-GCT-GAT-CAT-GGC-TGG-GTT-GT-3′ (antisense); *Ifn*-*r*, 5′-GAA AAT CCT GCA GAG CCA GA-3′ (sense) and 5′-TGA GCT CAT TGA ATG CTT GG-3′ (antisense); *Il*-*4*, 5′-TCA ACC CCC AGC TAG TTG TC-3′ (sense) and 5′-TGT TCT TCG TTG CTG TGA GG-3′ (antisense); *Grim19*, 5′-TCG-CCC-TTA-ATG-GTC-AGT-TC-3′ (sense) and 5′-CGA-GGA-GGA-TTT-TGA-GTG-TG-3′ (antisense); and regulator of calcineurin 3 (*Rcan3*), 5′-AGC–AGC-TGT-GTC-AGA-TGG-TG-3′ (sense) and 5-’CTG-AGC-AGT-CCC-CTG-TAA-GC-3′ (antisense). All transcriptional levels were normalized to that of β-actin.

### Flow cytometry

Mononuclear cells were immunostained with various combinations of fluorescent antibodies against CD4, CD25, FOXP3, IFN-γ, IL-4, IL-6, and IL-17 (eBioscience, San Diego, CA, USA). Prior to intracellular staining, cells were restimulated with phorbol myristate acetate (PMA; 25 ng/mL) and ionomycin (250 ng/mL) in the presence of GolgiSTOP (BD Biosciences) for 4 h. Intracellular staining was conducted using a kit (eBioscience) following the manufacturer’s protocol. Flow cytometry analysis was performed on a FACSCalibur flow cytometer (BD Biosciences).

### Elisa

The protein levels of IL-1β, IL-6, IL-17 and TNF-α in serum or culture supernatants were measured using sandwich ELISA (Duoset; R&D Systems, Lille, France). Serum levels of IgG and IgG1 antibodies were measured using a commercially available ELISA kit (Bethyl Laboratories, Montgomery, TX, USA).

### Staining for confocal microscopy analysis

Spleen tissue was obtained 14 days after BMT, snap-frozen in liquid nitrogen, and stored at −80 °C. Tissue cryosections (7 μm thick) were fixed with acetone and stained with FITC-, PE-, PerCP-Cy5.5-, or APC- conjugated monoclonal antibodies against mouse CD4, pSTAT3 (Tyr 705, Ser 727), pSTAT5, NFATc1, RCAN3, PD-1, CTLA-4, IFN-γ, IL-4, IL-17, and FOXP3 (eBioscience). After incubation overnight at 4 °C, stained sections were visualized by confocal microscopy (LSM 510 Meta; Zeiss, Göttingen, Germany). Immunohistochemistry was performed using Vectastain ABC kit (Vector Laboratories, Burlingame, CA, USA). Tissues were incubated with primary mouse anti-RCAN3 antibody overnight at 4 °C. Primary antibodies were detected using a biotinylated secondary antibody followed by incubation with streptavidin-peroxidase complex for 1 h. DAB chromogen was added (Dako, Carpinteria, CA, USA) to obtain the final colored product. Positive cells were counted and results were expressed as mean ± SD.

### Immunohistochemistry

Immunohistochemistry was performed using the Vectastain ABC kit as mentioned earlier. Tissues were incubated with primary anti-c-Jun and anti-c-Fos antibodies overnight at 4 °C. The primary antibody was detected with a biotinylated secondary antibody followed by incubation with a streptavidin-peroxidase complex for 1 h. DAB chromogen was added to obtain colored product.

### Murine T cell isolation and differentiation

To purify splenic CD4^+^ T cells, splenocytes were incubated with anti-CD4-coated magnetic beads. CD4^+^ T cells were isolated using magnetic-activated cell sorting (MACS) separation columns (Miltenyi Biotec). Cells were cultured in the presence of plate-bound anti-CD3 and soluble anti-CD28 (each at a concentration of 1 µg/mL; both from BD PharMingen), anti-interferon γ (anti-IFN-γ), and anti-IL-4 (each at a concentration of 5 µg/mL) for 3 days. Th17 cell differentiation was induced by treatment with IL-6 (20 ng/mL) and transforming growth factor β (TGF-β; 2 ng/mL). The cells were treated by STAT5 inhibitor (Santa Cruz) to reduce STAT5 expression.

### Transfection

To generate GRIM19 overexpression vector, *Grim19* fragment was synthesized by TOPgene Technologies (Quebec, Canada) with codon optimization for expression in mammalian cells. It was subcloned into the *Bam*H1 and *Xho*1 sites of pcDNA3.1(+) (Invitrogen). Mock and GRIM19 vector constructs were transfected using Amaxa 4D-Nucleofector X unit (Lonza, Switzerland) according to the manufacturer’s recommendations with the program DN-100 and P3 primary cell solution.

### RNA interference

To knock down the expression of GRIM19, CD4^+^ T cells were purified and nucleoporated with siRNA specific for Grim19 or scrambled siRNA (Santa Cruz Biotechnology, Santa Cruz, CA, USA), using an Amaxa Nucleoporator system. Briefly, 5–10 × 10^6^ CD4^+^ T cells were resuspended in 100 μL nucleofector solution and transfected with 100 nM siRNA using the U-014 Amaxa nucleofector program (Lonza, Switzerland). After transfection, cells were incubated at 37 °C for 6 h and stimulated with anti-CD3/CD28-coated magnetic beads under Th17-polarizing conditions for 48 h.

### Statistical analysis

Statistical analysis was performed with nonparametric Mann–Whitney *U* test using IBM SPSS Statistics 20 for Windows (IBM Corp., Armonk, NY, USA). *p* < 0.05 was considered as statistically significant. Data were presented as mean ± standard deviation (SD).

## Results

### GRIM19 inhibits alloreactive T cell response both in vitro and in vivo

**Fig. 1:**
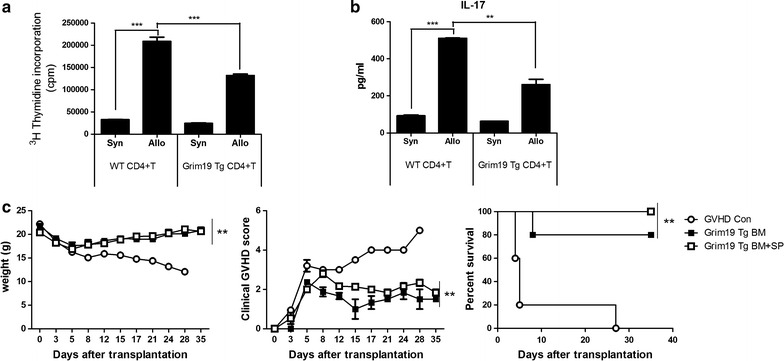
GRIM 19 blocks alloreactive T cell responses both in vitro and in vivo. **a** In the mixed lymphocyte reaction (MLR) assay, a total of 10^5^ RBC-lysed B6 or GRIM19 splenic T cells (responders) were incubated with 10^5^ irradiated RBC-lysed Balb/c splenic APCs (allogeneic [allo] stimulators) or B6 splenic APCs (syngeneic [syn] stimulators) for 4 days. T cell proliferation was measured through ^3^[H]-TdR incorporation. **b** IL-17 levels were determined in supernatants using ELISA. Data represent the mean ± SD of triplicate experiments. Data are representative of at least three independent experiments. **c** Recipients (B/c mice) were intravenously injected with 5 × 10^6^ donor (WT or GRIM19 Tg) bone marrow cells and 1 × 10^7^ splenocytes after lethal irradiation. Data are representatives of at least three independent experiments. GVHD clinical manifestations after allogeneic BMT were scored for weight loss, posture, activity, fur texture, and skin integrity. The combined data from two independent experiments (n = 20 per group). **p < 0.01 compared to GVHD control

As GVHD is induced by the activation of host-reactive donor T cells, persistence of alloreactive T cells is required for the development of GVHD [18]. Therefore, we first investigated whether GRIM19 could reduce alloreactive T cell response. In a mixed lymphocyte reaction (MLR), CD4^+^ T cells from B6 WT or GRIM19 Tg mice were cultured with allogeneic APCs. The alloreactive T cell response observed through the number of GRIM19 CD4^+^ T cells was decreased significantly (*p* < 0.05) compared to that with WT CD4^+^ T cells (Fig. 1a). In addition, IL-17 expression by GRIM19 CD4^+^ T cells was significantly (*p* < 0.05) reduced compared to that by WT CD4^+^ T cells (Fig. 1b). To verify the function of GRIM19 in inhibiting alloreactive T cell response, bone marrow (BM) and spleen (SP) from GRIM19 Tg mice were transplanted into recipient mice. BM and BM + SP groups had significantly (*p* < 0.05) higher survival rate than that of controls. The GVHD clinical score was also improved after the transplantation of BM or BM + SP from GRIM19 Tg mice, including no weight loss (Fig. 1c).

### GRIM19 has therapeutic activity of improving acute GVHD severity and inflammation

**Fig. 2:**
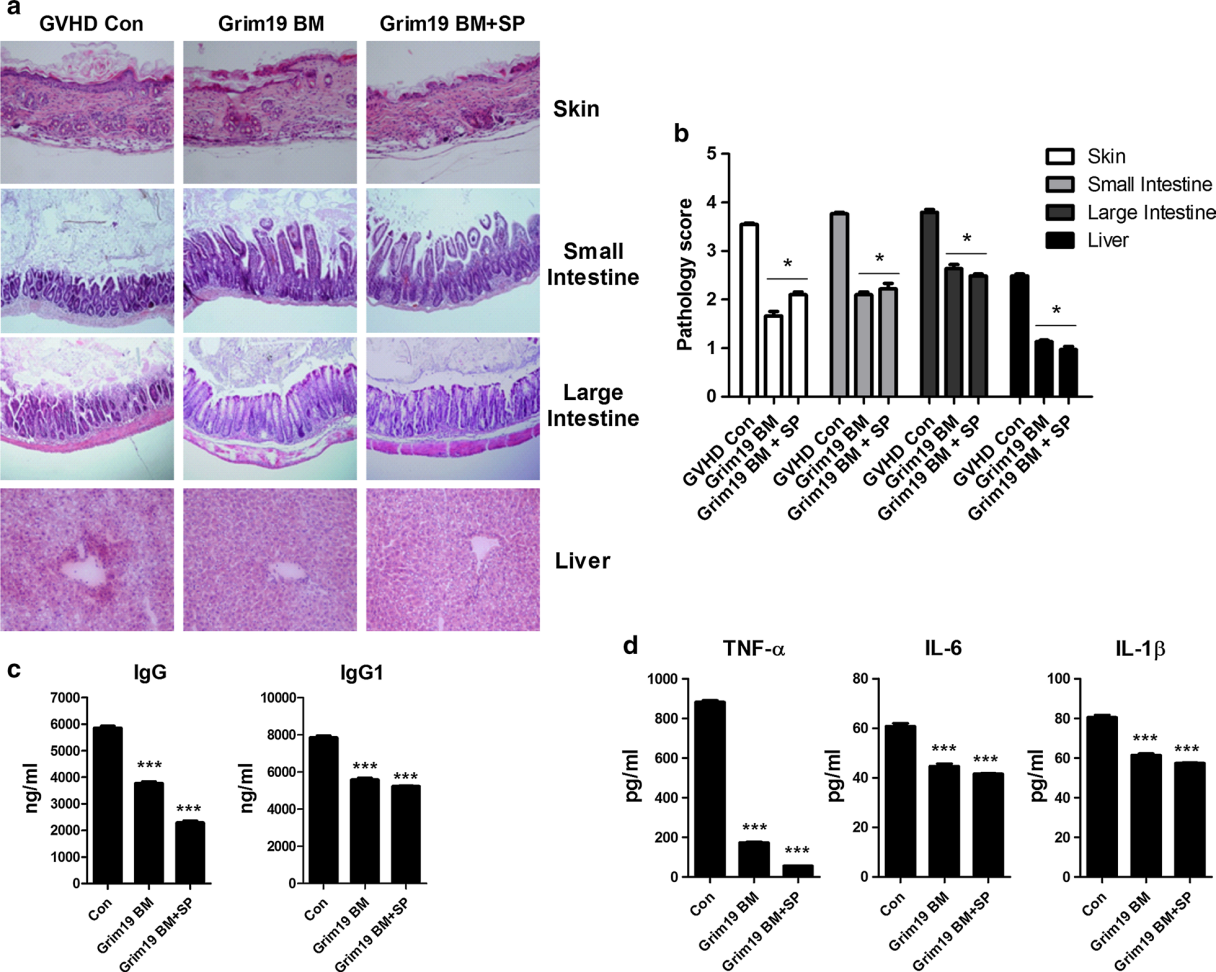
GRIM19 attenuates acute GVHD severity and inflammation. **a** Recipients (B/c mice) were intravenously injected with 5 × 10^6^ donor (WT or GRIM19 Tg) bone marrow cells and 1 × 10^7^ WT or GRIM19 Tg splenocytes after lethal irradiation. Histopathology results of the skin, liver, small intestine, and lung on day 14 after BMT (n = 12 per group) from one of two independent experiments are shown. Sections were stained with H&E (original magnification, ×200). **b** Histopathology score of the skin, liver, small intestine, and lung tissues. Results are expressed as mean ± SD from six mice. *p < 0.05, **p < 0.01. **c** Twelve days after BMT, serum levels of IgG in the three groups were measured by ELISA. Data are expressed as means ± SD from eight mice. *p < 0.05, **p < 0.01, ***p < 0.001. **d** Serum levels of IFN-γ, IL-17, and IL-10 in three groups were measured by ELISA. Data are expressed as means ± SD from eight mice. *p < 0.05, **p < 0.01, ***p < 0.001

Acute GVHD primarily affects skin, gastrointestinal tract, and liver [19–22]. Therefore, histopathology analysis was performed for intestinal, skin, and liver tissues to determine whether GRIM19 could reduce the severity of GVHD. The pathology score of the BM or BM + SP group was significantly (*p* < 0.05) lower than that of the control (Fig. 2a, b). Since GVHD is mediated by immune inflammation, we also measured IgG and proinflammatory cytokines concentrations. Both IgG and IgG1 levels were significantly (*p* < 0.05) reduced by the transplantation of BM or BM + SP from GRIM19 Tg mice. TNF-α, IL-1β, and IL-6 production in both GRIM19 Tg BM and BM + SP groups were lower than that in the control (Fig. 2c, d). These results showed that transplantation of BM or BM + SP derived from GRIM19 Tg mice attenuated the severity of GVHD.

### GRIM19 regulates T helper cell response

**Fig. 3:**
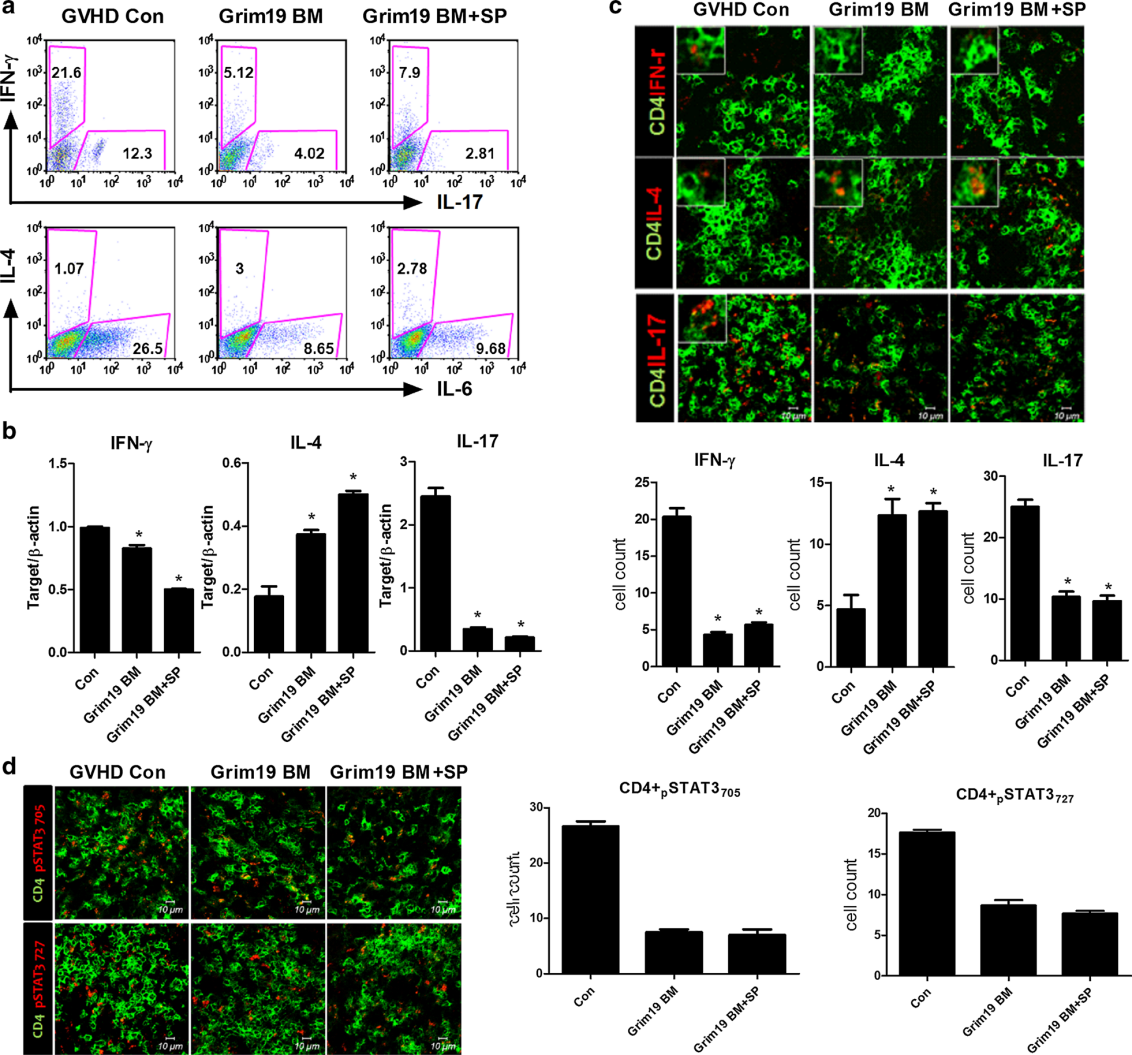
GRIM19 regulates T helper cell responses. **a** Recipients (n = 6 B/c mice per group) were intravenously injected with 5 × 10^6^ donor (WT or GRIM19 Tg) bone marrow cells and 1 × 10^7^ WT or GRIM19 Tg splenocytes after lethal irradiation. Intracellular cytokines were detected in CD4^+^ T cells. Fourteen days after BMT, splenocytes were isolated and the expression levels of IL-4, IL-17, and IFN-γ were determined by flow cytometry. **b** The mRNA levels of *Il*-*17*, *Il*-*4*, Treg, and *Ifn*-*γ* were analyzed by quantitative PCR. **p < 0.01. **c** Fourteen days after BMT, splenocytes were isolated and stained with anti-CD4 followed by reacting with anti-IL-17, anti-IL-4, and anti-IFN-γ antibodies. The expression patterns were visualized by confocal microscopy. **d** Expression levels of intracellular signaling molecules p-STAT3 (Tyr705) and p-STAT3 (Ser727) in splenic CD4^+^ T cells were determined by confocal microscopy on day 14 after allogeneic BMT. Confocal microscopy images were obtained for each mouse (n = 6 per group) and representative images are shown. *Bars* represent the mean ± SD from six mice per group. *p < 0.05, **p < 0.01. Data are representative of at least three independent experiments

GVHD is characterized by the differentiation of T cells present in the graft [1]. Excessive production of cytokines such as IL-1β, IL-6, IL-17, and IFN-γ by differentiated T cells can lead to inflammatory response and cause damage to several host tissues in GVHD [23]. In order to examine whether GRIM19 could regulate Th cell response, we analyzed Th cell subsets in the spleen. The expression of IL-1β, IL-6, IL-17, and IFN-γ were decreased in splenic CD4^+^ T cells from GRIM19 tg BM and BM + SP groups. The transcriptional levels of *Il*-*1β*, *Il*-*6*, *Il*-*17*, and *Ifn*-*γ* in splenocytes from GRIM19 tg BM and BM + SP groups were also significantly (*p* < 0.05) lower than those in the controls. On the other hand, the protein level of IL-4 was increased in splenic CD4^+^ T cells from GRIM19 tg BM and BM + SP groups. The transcriptional levels of *Il*-*4* in splenocytes from GRIM19 tg BM and BM + SP groups were also significantly (*p* < 0.05) higher than those in the controls (Fig. 3a, b). Confocal microscopy analysis revealed that the number of CD4^+^ T cells producing IL-17 and IFN-γ was significantly (*p* < 0.05) decreased. However, the number of CD4^+^ T cells expressing IL-4 in splenocytes from the GRIM19 tg BM and BM + SP groups was increased significantly (*p* < 0.05) compared to that of the controls (Fig. 3c).

STAT3 is an important transcription factor for Th17 differentiation [24]. Therefore, we evaluated the expression of STAT3. The number of cells expressing activated form of STAT3, p-STAT3 Tyr705, and Ser727 was reduced significantly (*p* < 0.05) among splenic CD4^+^ T cells from GRIM19 tg BM and BM + SP groups (Fig. 3d).

### GRIM19 induces Tregs by promoting STAT5 expression

**Fig. 4:**
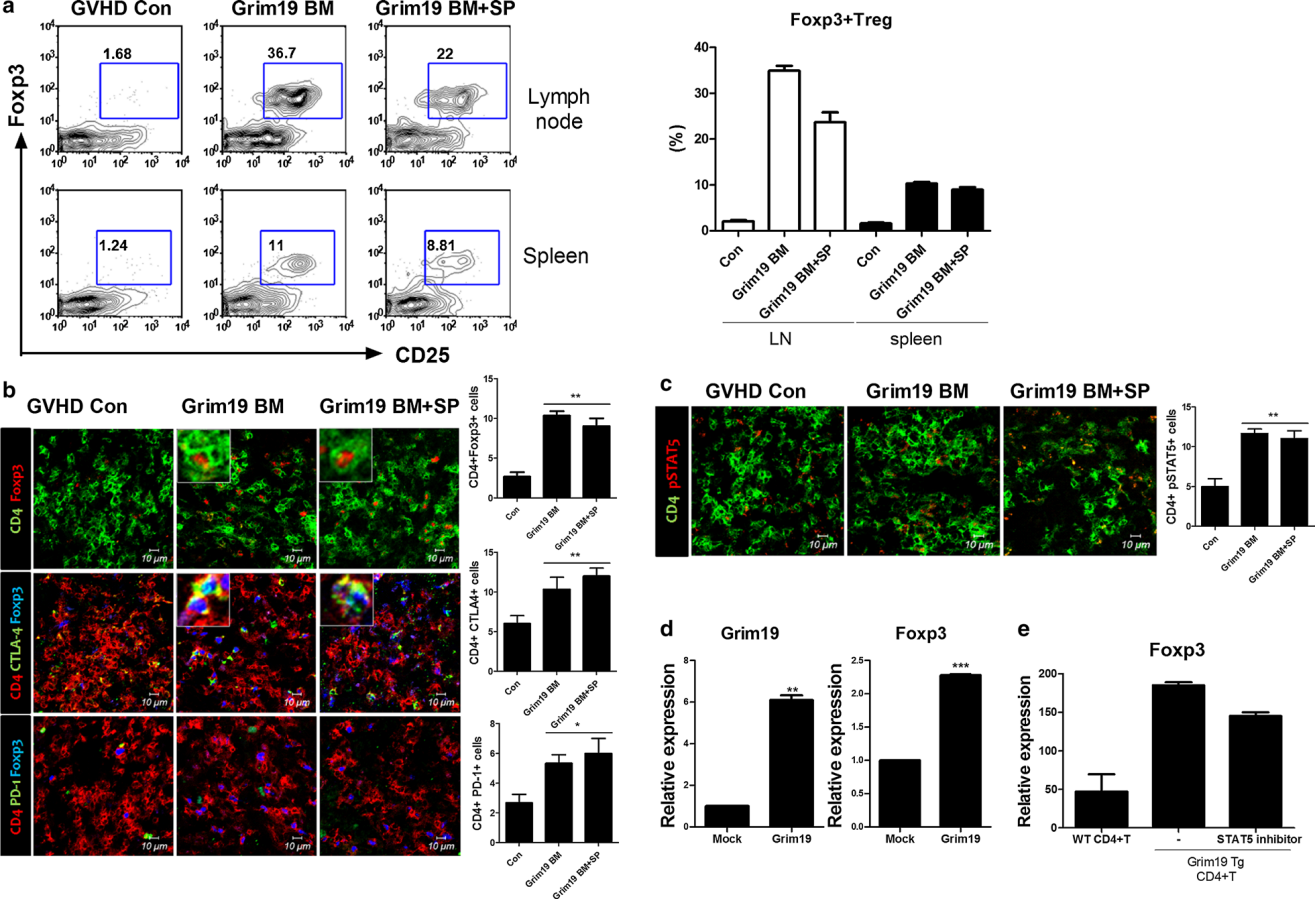
GRIM19 regulates Treg/Th17 ratio via inhibiting STAT3 pathway. **a** Recipients (B/c mice) were intravenously injected with 5 × 10^6^ donor (WT or GRIM19 Tg) bone marrow cells and 1 × 10^7^ WT or GRIM19 Tg splenocytes after lethal irradiation. Intracellular immunostaining was performed for FOXP3^+^ Treg (CD4^+^CD25^+^) cells in spleen and LN. Data in the* left panel* are representatives of three independent experiments.* Bars* show the mean ± SD of at least three independent experiments. **b** Fourteen days after BMT, isolated spleens were analyzed by confocal microscopy for the expression of GITR and PD-1 among CD4^+^CD25^+^Foxp3^+^ regulatory T cells. *Bars* represent the SD of six mice per group. **c** The expression of signal transducer phosphorylated STAT5 in the spleens was analyzed by confocal microscopy on day 14 after BMT. Data are representatives of three independent experiments. **d** Splenic CD4^+^ T cells were sorted from B6 mice and transfected with mock or GRIM19 vector. Mock or GRIM19-transfected T cells were activated by stimulation with anti-CD3**/**CD28 for 3 days. *Grim19* and *Foxp3* mRNA levels were determined by real-time PCR. Data are expressed as mean ± SD of three independent experiments. *p < 0.05, **p < 0.01 *vs*. mock. **e** Splenic CD4^+^ T cell isolated from WT or GRIM19 Tg mice were activated by stimulation with anti-CD3**/**CD28 for 3 days. A STAT5 inhibitor was added at priming. *Foxp3* mRNA level was determined by real-time PCR. Data are expressed as mean ± SD of three independent experiments

Cytotoxic T-lymphocyte antigen (CTLA)-4 and programmed death (PD)-1 receptor are expressed on the surface of activated T cells [25]. Tregs, a subset of T cells, have immunosuppressive functions. They also express CTLA-4 and PD-1. Their differentiation is regulated by STAT5 [24, 26]. Reduced number of Tregs is known to be a key factor in GVHD pathophysiology [27]. In addition, Treg/Th17 ratio is a specific indicator of human GVHD [28]. Therefore, we measured the expression levels of CTLA-4, PD-1, FOXP3, and STAT5 in the spleen and lymph nodes of mice with GVHD. FOXP3 expression was found to be increased in the spleen and lymph nodes of GRIM19 tg BM and BM + SP groups (Fig. 4a). The number of Treg cells expressing CTLA-4, PD-1, and pSTAT5 was also increased in the spleens of GRIM19 tg BM and BM + SP groups (Fig. 4b, c). Next, we determined whether GRIM19 expression might play a role in Treg differentiation. To test whether GRIM19 could induce FOXP3 expression, we transfected murine CD4^+^ T cells with a GRIM19 overexpression vector. The transcriptional levels of *Grim19* and *Foxp3* were significantly (*p* < 0.05) higher in GRIM19-overexpressing CD4^+^ T cells compared to those in mock vector group (Fig. 4d). We also investigated the interaction between *Grim19* and *Foxp3*. The transcriptional level of *Foxp3* gene in CD4^+^ T cells from GRIM19 Tg mice was higher than that from WT CD4^+^ T cells. Moreover, the transcriptional level of *Foxp3* in GRIM19 Tg CD4^+^ T cells was significantly (*p* < 0.05) reduced by STAT5 inhibitor (Fig. 4e).

### IL-17 is inhibited by reduced STAT3-NFATc1 expression

**Fig. 5:**
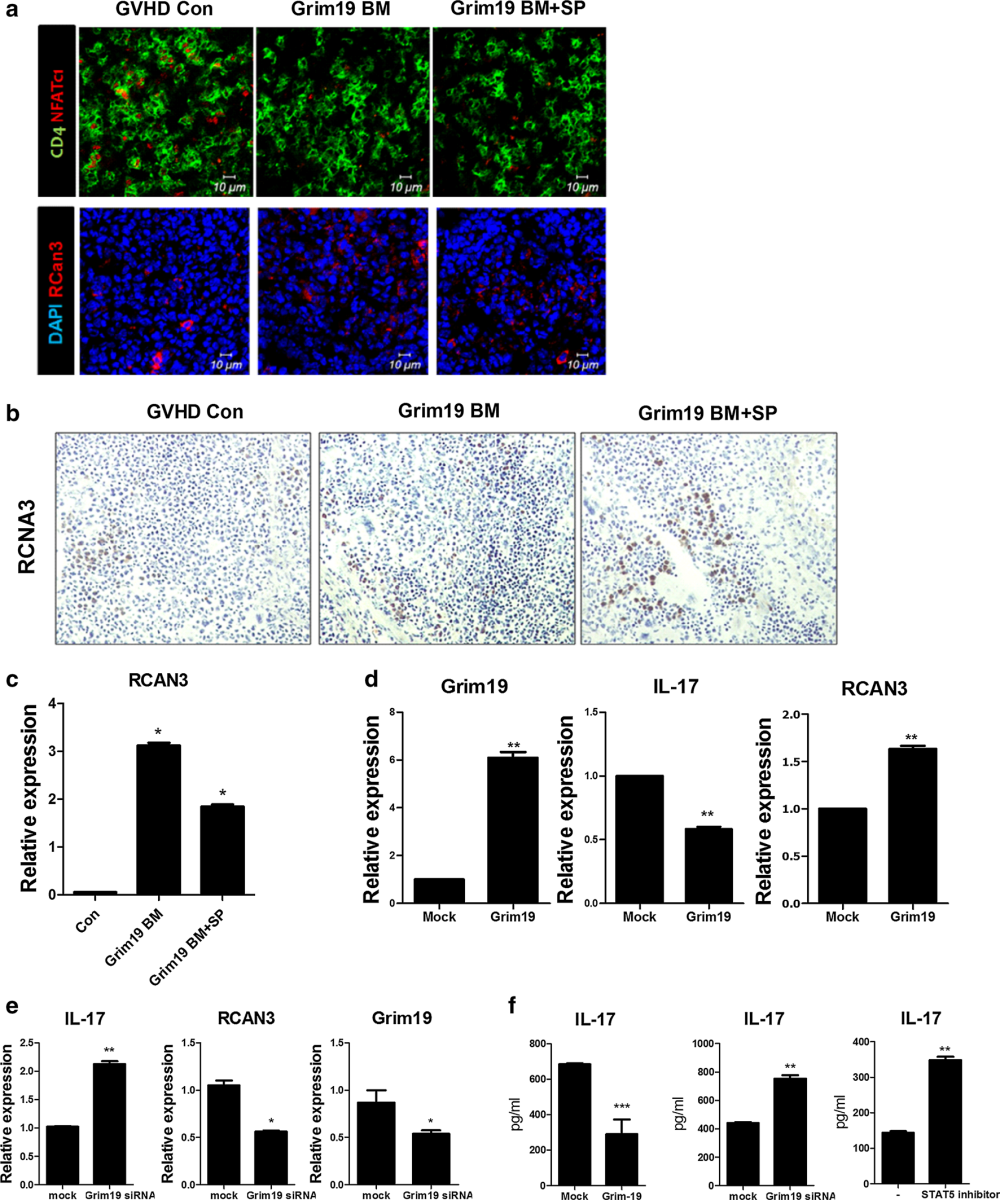
GRIM19 inhibits IL-17 by downregulating STAT3-NFATc1. **a** Recipients (B/c mice) were intravenously injected with 5 × 10^6^ donor (WT or GRIM19 Tg) bone marrow cells and 1 × 10^7^ WT or GRIM19 Tg splenocytes after lethal irradiation. The expression levels of intracellular signaling molecules p-NFATc1 and RCNA3 in the spleen were determined by confocal microscopy on day 14 after allogeneic BMT. All confocal microscopy images were obtained for each mouse (n = 6 per group). Representative images are shown. **b** Immunohistochemical staining for RCAN3 in spleen tissue from GVHD mice. Positive immunoreactivity appears as a brown color. It is counterstained with blue or green. Original magnification, ×400. **c** Fourteen days after BMT, *Rcan3* mRNA levels in splenocytes were determined by real-time PCR. **d** Splenic CD4^+^ T cells from B6 mice were transfected with mock or GRIM19 expression vector. Mock or GRIM19-transfected CD4^+^ T cells were activated by stimulation with anti-CD3**/**CD28 (Th0 condition) for 3 days. *GRIM9*, *Il*-*17*, and *Rcan3* mRNA levels were determined by real-time PCR. **e** Splenic CD4^+^ T cells from B6 mice were transfected with mock or *Grim19* siRNA under Th0 conditions. *Il*-17 and *Rcan3* mRNA levels were determined by real-time PCR. **f** Splenic CD4^+^ T cells from B6 mice were transfected with GRIM19-overexpression vector, *Grim19* siRNA, or STAT5 inhibitor. IL-17 levels in supernatants were measured by ELISA. Data are expressed as mean ± SD of three independent experiments

Since NFATc1 and STAT3 cooperatively exacerbate inflammation [29] while RCAN3 inhibits the gene expression of NFAT-dependent cytokines [30], we measured the protein level of NFATc1 in the spleen of GVHD mice. NFATc1 protein level was decreased in the spleens of GRIM19 tg BM and BM + SP groups while the protein level of RCAN3 was increased (Fig. 5a, b). The transcriptional level of *Rcan3* mRNA was also increased significantly (*p* < 0.05) in the spleens of GRIM19 tg BM and BM + SP groups compared to that in the controls (Fig. 5c). Next, we transfected murine CD4^+^ T cells with a GRIM19 overexpression vector to verify whether GRIM19 could regulate the transcriptional level of *Il*-*17* and *Rcan3*. Our results revealed that the transcriptional level of *Il*-*17* was reduced significantly (*p* < 0.05) while the transcriptional level of *Rcan3* was increased significantly (*p* < 0.05) in GRIM19-overexpressing cells compared to that in mock vector-transfected cells (Fig. 5d). To confirm that GRIM19 could regulate the expression of *Il*-*17* and *Rcan3*, we evaluated the mRNA expression levels of *Il*-*17* and *Rcan3* in murine CD4^+^ T cells treated with *Grim19* siRNA. Treatment with *Grim19* siRNA significantly (*p* < 0.05) reduced the transcriptional level of *Rcan3* gene. However, the transcriptional level of *Il*-*17* was significantly (*p* < 0.05) increased after *Grim19* siRNA treatment (Fig. 5e). We also studied the effect of GRIM19 on the production of IL-17. Based on ELISA, IL-17 production was decreased (*p* < 0.05) significantly by GRIM19 overexpression. On the other hand, *Grim19* siRNA and STAT5 inhibitor significantly (*p* < 0.05) upregulated the protein level of IL-17 (Fig. 5f).

## Discussion

The role of GRIM19 in apoptosis has been studied extensively [10]. Recently, GRIM19 has also been found to be involved in inflammatory diseases and T cell-mediated immune responses. However, little is known about the mechanism of GRIM19 activity in GVHD. Here, we investigated the therapeutic function of GRIM19 in GVHD and identified a new mechanism for GVHD modulation.

The most remarkable finding of this research was that GRIM19 attenuated GVHD via downregulating alloreactive T cell response. To our knowledge, this is the first study that provides evidence suggesting that GRIM19 could be used as a therapeutic agent to treat GVHD. Previous reports have demonstrated that alloreactive T cell responses contribute to GVHD [18, 31]. In addition, it has been suggested that IL-17 can increase the severity of GVHD by activating CD4^+^ T cells [32]. Our results indicated that GRIM19 could inhibit alloreactive T cell responses and IL-17 production well known to be able to reduce GVHD severity, suggesting a novel therapeutic strategy to modulate GVHD via GRIM19.

GVHD affects several tissues, including the skin, liver, and intestine that are damaged in acute GVHD [19–22]. As GVHD is an immune inflammatory disease, IgG expression [33, 34] and levels of inflammatory mediators such as IL-1β, IL-6, and TNF-α is increased in patients with GVHD [35–38]. In this study, transplantation of BM and SP from GRIM19 Tg mice was found to be able to improve the pathological status. Moreover, the production of IgG and proinflammatory cytokines that could induce the classical cytokine storm were significantly reduced in GRIM19 Tg groups. These results suggest that GRIM19 can reduce GVHD severity by inhibiting IgG and proinflammatory cytokine productions.

Several subsets of T cells take are involved in the pathogenesis of GVHD. Indeed, the production of cytokines including IFN-γ by Th1 cells [39, 40] and IL-17 expression by Th17 cells contributes to GVHD morbidity [32]. By contrast, inhibition of Th17 differentiation significantly reduced tissue pathology in the liver and intestine [41]. Moreover, Th1 and Th17 cells can accelerate the development of GVHD [42] whereas Th2 cells revealed protective function in GVHD [39], and attenuated the GVHD severity through IL-4-mediated mechanism [43]. Additionally, the immunosuppressive function of Tregs plays a pivotal role in the suppression of GVHD [27, 44]. Mechanistically, T cell subset differentiation is regulated by different transcription factors. Indeed, Th17 cell differentiation is regulated by STAT3, while Treg differentiation is controlled by STAT5 [24, 42]. In patients with GVHD, p-STAT3 Tyr 705 is enhanced significantly. In addition, Th17 cells are associated with GVHD severity [45]. Conversely, STAT5 can induce Treg differentiation and limit human T cell alloresponses [46, 47]. Our results revealed that GRIM19 could inhibit canonical function of STAT3 regulating the expression of proinflammatory cytokines. These data indicate that GRIM19 has therapeutic function for GVHD based on the regulation of Th17/Treg balance through suppressing STAT3 expression and inducing STAT5 expression.

NFATc1 is a member of the NFAT family of transcription factors involved in T cell activation. Several researchers have reported that STAT3 activation can increase the expression of NFATc1 and that the interaction between STAT3 and NFATc1 will aggravate inflammation [29, 48]. Moreover, suppression of NFATc1 can improve inflammatory diseases [49, 50]. RCAN3 is a calcineurin inhibitor like FK506 and can reduce cytokine mRNA levels associated with NFAT [30]. In this study, the transplantation of BM and SP from GRIM19 Tg mice downregulated the expression of NFATc1 but induced the production of RCAN3. GRIM19 overexpression also decreased gene expression of *Il*-*17* and the production of IL-17 cytokine. However, it increased the mRNA level of *Rcan3*. In addition, the inhibition of GRIM19 and STAT5 increased the expression level of *Il*-*17* mRNA and IL-17 protein but reduced the mRNA expression of *Rcan3*. Thus, GRIM19 could decrease inflammation via inhibiting the STAT3-NFATc1 axis.

There are some limitations of this study. First, this study had a relatively short-term follow up of GVHD in several in vitro assays and in vivo tests that demonstrated attenuation in GVHD severity. We did not show the activity of GRIM19 in GVHD with a long-term follow up. Further study is merited to follow-up the GVHD mouse model for longer time to confirm the function of GRIM19. Clinical application using Grim19 overexpression to suppress T cell activation and inflammation is also needed to confirm the translational potential of GRIM19 for treating GVHD. Another limitation of this study was the measurement of GVL activity. Generally, inhibition of alloreactive T cells can effectively prevent GVHD. However, it usually leads to reduced GVL activity which can appear after allogeneic hematopoietic stem cell transplantation. Although knowing the mode of action of GRIM19 is important to effectively treat GVHD, the mechanism of RCAN3 is not revealed in this study. Further studies are required to investigate the translational potential of Grim19 overexpressed cells and determine the mechanisms involved in RCAN3 action.

## Conclusion

Our findings suggest a therapeutic effect of GRIM19. It will shed new light on the treatment of GVHD. We demonstrate that GRIM19 inhibits the canonical STAT3 pathway and controls reciprocal regulation Th17/Treg. This empirical evidence demonstrates that GRIM19 is a promising candidate for treating GVHD.
